# Toll free mobile communication: overcoming barriers in maternal and neonatal emergencies in Rural Bangladesh

**DOI:** 10.1186/1742-4755-11-52

**Published:** 2014-07-12

**Authors:** Nafisa Lira Huq, Asrafi Jahan Azmi, MA Quaiyum, Shahed Hossain

**Affiliations:** 1 International Centre for Diarrheal Disease Research, Bangladesh (icddr, b). 68, Shahid Tajuddin Ahmed Sarani, Mohokhali, Dhaka 1212, Bangladesh

**Keywords:** Toll free mobile phone, Maternal health complications, Rural Bangladesh

## Abstract

**Background:**

Toll free mobile telephone intervention to support mothers in pregnancy and delivery period was tested in one sub district of Bangladesh. Qualitative research was conducted to measure the changes of mobile phone use in increasing communication for maternal and neonatal complications.

**Methods:**

In-depth interviews were conducted among twelve Community Skilled Birth Attendants and fourteen mothers along with their husbands prior to intervention. At intervention end, six Community Skilled Birth Attendants were purposively selected for in-depth interview. Semi structured interviews were conducted among all 27 Community Skilled Birth Attendants engaged in the intervention. One Focus Group Discussion was conducted with 10 recently delivered mothers. Thematic analysis and triangulation of different responses were conducted.

**Results:**

Prior to intervention, Community Skilled Birth Attendants reported that mobile communication was not a norm. It was also revealed that poor mothers had poor accessibility to mobile services. Mothers, who communicated through mobile phone with providers noted irritability from Community Skilled Birth Attendants and sometimes found phones switched off. At the end of the project, 85% of mothers who had attended orientation sessions of the intervention communicated with Community Skilled Birth Attendants through mobile phones during maternal health complications. Once a complication is reported or anticipated over phone, Community Skilled Birth Attendants either made a prompt visit to mothers or advised for direct referral. More than 80% Community Skilled Birth Attendants communicated with Solution Linked Group for guidance on maternal health management. Prior to intervention, Solution Linked Group was not used to receive phone call from Community Skilled Birth Attendants. Community Skilled Birth Attendants were valued by the mothers. Mothers viewed that Community Skilled Birth Attendants are becoming confident in managing complication due to communication with Solution Linked Group.

**Conclusions:**

The use of mobile technology in this intervention took a leap from simply rendering information to providing more rapid services. Active participation of service providers along with mothers’ accessibility motivated both the service providers and mothers to communicate through mobile phone for maternal health issues. These altogether made the shift towards adoption of an innovation.

## Background

According to the Bangladesh maternal mortality and health care survey, 2010, the estimated maternal mortality ratio (MMR) in Bangladesh is 194/100,000 live birth [1]. There are many factors responsible for maternal death in Bangladesh; the factors are more than 80% of deliveries are conducted at home with untrained birth attendants [2], delays in recognition of maternal complications [3] and limited referral linkages and transportation for emergency obstetric care [4,5]. In addition, lack of knowledge on maternal and newborn danger signs could delay the access to health care during maternal health complications and delayed access to care affect poor birth outcomes [6]. Studies also suggested that the probability of delay in access to care is due to inadequate understanding about the need for immediate care from qualified providers, financial and transport barriers [7]. Birth outcomes are once more affected due to lack of skilled providers; World Health Organization (WHO) country cooperation strategy 2008–2013, Bangladesh [8] reported that Bangladesh continues to face a chronic shortage and imbalance in the health work force skill mix for maternal and neo-natal health. Consequently, health interventions need to formulate alternative strategies in improving maternal health outcomes. Health care telephonic advice is recommended as one of the possible solutions to the low income countries in order to address these gaps [9].

High availability of mobile phones in Lothian and Kent cities of United Kingdom is encouraging patients’ compliance in chronic disease like asthma [10]. Projects in, Malawi, Uganda, Ghana and Sierra Leone equipped the Traditional Birth attendants (TBAs) and /or midwives with walkie talkie. The TBAs utilized this system in contacting skilled supervisors and ambulances during complicated situations and noted a significant reduction in maternal deaths [11-14]. Mobile phone was provided to TBAs and outreach workers by the emergency services departments in Gambia for referral of maternal complications. A project in Madhya Pradesh, India offered pregnant mothers a health telephone helpline, complimentary ambulance system, and drivers equipped with mobile phones [15]. Evaluation had not accounted for the impact of these projects; qualitative evidence from pilot programs in similar locations suggested reducing delays were associated with decision making and identifying appropriate health facility while introducing 24-hour obstetric mobile-phone-based helplines [15]. However, critical lessons learnt from the Uganda and Gambia projects was that maternal health outcomes were affected for unreliable emergency transport and poor quality of services at health facilities [15].

Projects on mhealth are mostly based on text messaging to pregnant mothers, reminding the pregnant mothers on their antenatal schedule or referral of high risk mothers [16]. However, either individual counseling or direct discussion over phone would be more preferable than receiving text messaging by some patients [17]. Other argued [18] that only access to communication tools is not adequate for decreasing maternal death in remote rural areas. Impact of interventions on reduction of maternal mortality should pay particular attention to the rapid accessibility to quality emergency obstetric care [19].

In Bangladesh, Grameen telephone through its micro credit program distributed mobile telephones with solar based battery rechargers to mothers living in rural villages. This provides them the direct access to agricultural commodity pricing and in addition, transfer of funds, access to medical services, contact with distant family members in family emergencies and medical situations etc. [20]. This is likely to acquire knowledge while mothers are equipped for better communication, but such program was not evaluated to understand its impact on reducing pregnancy related health risks.

An integrated maternal and neonatal health (MNH) intervention package focusing on health system strengthening, training of community skilled birth attendants, demand side financing, formation of community support groups was developed and tested in Shahjadpur, a sub district of Bangladesh by International Centre for Diarrheal Disease, Bangladesh (icddr, b). The MNH intervention was further benefited through integrating a toll free mobile phone intervention. The activities of the mobile phone intervention were implemented during September 2009 to March 2011. The effect of the intervention was measured through comparing results between baseline and endline survey (reported elsewhere). The intervention aimed to create demand in seeking skilled maternal health care service in particular maternal complications through establishing a toll free mobile pathway among mothers, Community Based Skilled Providers (CSBAs) and specialized maternal health providers.

Two qualitative researches on maternal health care seeking practice were conducted in two different time periods within this toll free mobile phone intervention. The formative research was undertaken prior initiating the toll free mobile phone intervention. This research explored mother’s accessibility to mobile communication, CSBA’s, mother’s and their husband’s perceived advantages and disadvantages of mobile phone communication for the first stage rapid management of maternal and neonatal complications. The formative research findings supported in finalizing the information needed for mother’s and CSBA’s participation in the intervention. At the end of the intervention another qualitative research was conducted which critically looked at care-seeking practices and important determinants for practices. Comparison between the two qualitative researches explored how attitudes and behaviors modified to increase demand for and timely use of health services through mobile communication. The baseline and endline surveys measured the effect size of the intervention; however it is also important to understand why there was change in behavior and also how the intervention worked within the changed behavior. This paper focused the attributes of the participants that supported to initiate the mobile phone pathway, explained the reasons to observe the changed behavior in this communication pathway before and after the intervention through the qualitative results.

## Methods

### Study site and intervention

Two qualitative researches were conducted at Shahjadpur sub-district of Sirajgonj district under the Rajshahi division. Shahjadpur is situated in the Northern part of Bangladesh, about 110 km northwest of Dhaka, the capital city of Bangladesh. The sub district has an estimated population of 583,350 according to the 2011 Bangladesh national census. Shahjadpur comprises of female population within the reproductive age group (15–49 years) of 150,000. This sub district is mainly on an agricultural economy with a large number of cottage-based weaving factories. This sub district is divided into 13 unions (lowest administrative unit in rural areas) and 01 municipality. Shahjadpur has a 50 bed primary public health care centre, several private clinics that offer cesarean section and 62 CSBAs (in public and private employment), covering approximately the requirement of 01 CSBA per 8,000-10,000 population.

The toll free mobile phone intervention was a cluster-randomized controlled trial. The sub district was divided into two equal halves based by the 13 unions and 01 municipality area, individual union was considered the cluster. Thirteen unions and 01 municipality area were selected randomly and equal numbers of clusters (unions and municipality area) were assigned to the intervention and control areas.

Pregnant mothers were identified from a list routinely prepared by the Family Welfare Assistants (FWA) who works at community level under the Ministry of Health and Family Welfare (MOHFW). The field implementation team along with the respective CSBAs oriented the mothers, husbands and other family members like mother-in-law on the operational manual for the toll free mobile communication. At the same orientation sessions they provided information on the five danger signs during pregnancy, delivery and after delivery. The orientation session conducted at the yard of the pregnant mothers’s house or nearby suitable vicinity.

In the intervention unions mobile phones with a single designated number were provided to the respective CSBAs. The project personnel oriented the CSBAs on the technical use of toll free mobile communication. A mobile phone call log book was given to the CSBAs for recording the calls. Information on calls they received and actions taken were also noted in the log book.

An eight member Solution link Group (SLG) was formed at the Sub District level with expert medical persons from disciplines of obstetrics, gynecology, child health and nurse/midwifery. They were provided with mobile phones, orientated on the project activities and their expected participation in the mobile communication pathway. Their activities included:

a. Their role and support in the mobile communication pathway.

b. Response to a CSBA call in the terms of advises, referral and guidance.

c. Maintenance a call record book (Log Book).

Forty seven community support groups (CSGs) were formed each in a defined geographical and population area with the community stakeholders. CSGs were oriented about maternal and neonatal health emergencies, and on where and how to make a toll free call in emergencies. One CSG had 15–20 mothers volunteer members from the local area, supported by a 5 member advisory committee and 05–07 member executive committees. The CSGs were expected to disseminate maternal and neonatal health information and support families during maternal and neonatal health emergencies.

**Figure 1:**
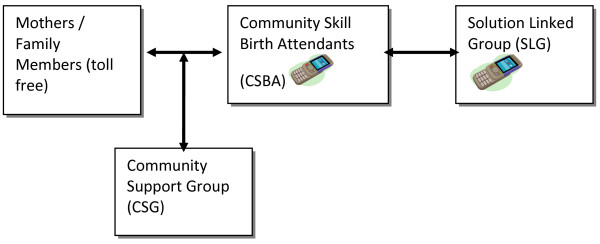
Mobile phone access pathway.

As shown in Figure 1[21], the first set of communication was between mothers / family members and the CSBAs. The mothers /family members were eligible to call free of charge for maternal and neonatal health and emergencies to a single designated number from any call operators to the local CSBA. The CSBAs would receive call from the mothers, provide advises or treatment by household’s visits, and make referrals if necessary.

In the second set of communication, the CSBAs could call the SLG members and seek advises from experts for conditions that they were unable to provide first hand care or advises. The SLG in turn would provide appropriate advises, instructions and if necessary referrals to appropriate places for care. Both the CSBAs and SLG members could call back the mothers or others if necessary in the course of managing a condition. All these calls were either free of charge or reimbursed.

### Participants’ recruitment and data collection

The formative research was conducted between January-February 2010 prior to introduction of the toll free mobile phone intervention. During the formative research, 12 in-depth interviews were conducted with the CSBAs. Fourteen mothers (7 pregnant mothers and 7 recently delivered mothers) along with their husbands participated in the in-depth interviews during the formative research. One husband declined to participate; no reasons were given for this declination. Therefore, a total of 14 mothers and 13 husbands were interviewed. The mothers were selected from different income groups - low income (monthly income less than 100 dollars), medium income (in between 100–200 dollar) and high income (equal or more than 200 dollars). The socioeconomic status was crudely assessed by income rather than using standardized items. The participants were selected purposively from the intervention unions using the available pregnant mother’s list.

The endline qualitative component was conducted in 2011 at the end of the intervention. In the endline, the FGD was conducted in one ‘Hard to Reach’ union and included 10 mothers (recently delivered) in the FGD. The ‘Hard to Reach’ area was defined on the distance between the villages and sub district health complex. Commonly the villages which are situated 10 km to 25 km away from the sub district health complex have poor road infrastructure and some of villages has no options for communication other than boat. In the semi structured interview 27 CSBAs were included. This number is the total number of CSBAs who participated in the intervention area. Six CSBAs of the intervention unions were selected purposively for the in-depth interview at the end of the intervention.

Mothers were interviewed at their homes. Husbands were interviewed either at home or at their workplace, depending on their availability. In-depth interviews of CSBA’s were conducted at their residence. The in-depth interview and the FGD lasted between 45 minutes and one hour. The semi structured interview required 15 minutes to complete. The interviews were audio-taped with participant’s permission and fully transcribed in Bangla.

Separate guidelines were used for in-depth interviews of mothers, husbands and CSBAs in this qualitative research. The guideline was developed based on the theoretical framework described below. Two anthropologists were trained on the guidelines which were developed in Bangla. After the two days class room training on the guideline the researchers went for a practice session in one of the clusters of Shahjadpur. The participants of the practice session did not include the actual implementation of the qualitative research. The results of the practice sessions supported to change the language of the questions and flow of the guidelines.

The guideline for the mothers included a small number of open ended questions to generate discussion which was followed by probing question to explicit the details. The questions of the guideline covered the obstetric history (parity, birth attendant at delivery and experience during maternal complications) and relating these to the communication process between mothers and providers. The communication process first started with the availability and accessibility of mobile phone for health care by mothers, their current use status of mobile phone for maternal and neonatal health problems, factors that facilitate or hinder the use of mobile phone and their willingness for future use of such communication. The FGD guideline in addition to these questions focused particularly on the mobile phone communication between CSBAs and SLGs, the benefits, obstacles and necessary actions taken due to such communication and suggestions to improve this communication pathway. The in-depth interviews with the CSBAs explored CSBAs’ willingness to communicate with the SLG to receive advice on maternal and neonatal care and suggestions to improve this communication. The semi structured interview included the characteristics of mothers who used this communication pathway to contact the CSBAs, the anticipated reasons for not communicating, the point of time for this communication during the childbirth period. Frequency of CSBAs communication with the SLG and the reasons for such communications were also explored in the in-depth interview at the endline.

### Data analysis

Transcripts were prepared in Bangla from the tape-recorded in-depth interviews. We conducted initial coding based on the several prior themes identified at the beginning of our research, for example, communication process, use of mobile phone, views of mobile communication in maternal health, recommendation for mobile communication. These prior themes were determined on the basis of the intervention aims and activities. Initial analysis was undertaken by the field researcher using conventional thematic coding techniques that identified and described the perspectives and experiences of participants. The process of coding continued until we reached the point of saturation that means there were no new code emerging and then we developed a code list. The coding list was finalized after the consensus agreement among the field researchers and investigators. Once this qualitative integrative analysis was completed we undertook a matrix framework procedure in which the similar codes were presented by respondent category. In this matrix we included key words or shortened quotations. This process helped in searching for patterns, associations, and explanations in the data. When possible, we triangulated responses under the different codes from different groups of respondents. Data analysis for the semi structured interview was done using Stata program 10.0 version for windows. Descriptive analysis was conducted for the semi structured interviews.

### Theoretical framework

Determinants for adoption of an innovation can be explained by Roger’s diffusion theory that illustrated how new ideas or innovations are disseminated and adopted among community [22]. According to Rogers, the diffusion of an innovation is enhanced when the perceived superiority of an innovation is high compared to existing practice (i.e. the relative advantage). Low complexity of an innovation and adopted on a trial basis showed easily observed results, being associated with greater adoption and swifter diffusion. Building success and comfort during the early stages of the implementation of an innovation will be paramount to its success. According to this theory, the relevant factors for successful mobile communication pathway in rapid management of maternal complication are participants’ predisposition to mobile phone communication, their perceived advantages of this pathway and the infrastructure that would enable them to participate in this innovation. In the context of the present study, all these factors had been taken into consideration on which in-depth information was collected.

### Ethical considerations

The research was conducted after receiving IRB approval from icddr, b. Written consent was obtained from each participant after explaining the purpose of the study, information to be collected and risk and benefit for participating in the research. A tape recorder was used to record the in-depth interview and FGD with participant’s permission.

## Results

### Demographic data

The age range of mothers included in the formative research was 20 to 25 years among 08 mothers, two mothers were below 20 years of age and four were in their early thirties. Most of the mothers were Muslim. Four of them stated that they had no formal education; the remaining 10 mothers completed primary or secondary school (Grade 5 to 10). Eight mothers had <5 km distance from the sub district health complex, 05 mothers had this distance up to 10 km and 01 lived 25 km away from the health complex. Seven mothers were living in a family who had a monthly income between $100 to 200, for four mothers this was > $200 and for another four mothers it was < $100. The 10 recently delivered mothers included in the FGD at the endline were aged between 20–25 years. Majority of them had no formal education. All the mothers included in the FGD were residing in a union which was 10 km away from the health complex and living in a family with a monthly income between $100-200. The CSBAs included in the intervention had secondary level of education and all were married. The average age of the CSBAs was 35 years (range: 25–40 years).

### Communication and accessibility by mobile phone with CSBAs

At the end of the intervention, nearly 90% CSBAs reported in the semi structured interview that mothers communicated through mobile phone with them for maternal health issues. ‘Mothers went to their parental house for delivery’ was the common reason for non communication through phone with CSBA during delivery. CSBAs also mentioned that mother’s preference for TBAs as birth attendants, rich mother’s preference for facility care and absence of maternal complications were other reasons for non communication. At the end of the project, 85% of mothers who had attended orientation sessions of the intervention communicated with CSBAs through mobile phones during maternal health complications. CSBAs also indicated that mothers received messages about the mobile phone intervention from their relatives, respective CSBAs and CSG (Table 1).

**Table 1:** Communication through toll free mobile phone with the CSBAs as reported by CSBAs

**Communication**	**Percentage (n = 27)**
Communication for maternal issue	88.7
No communication	11.3
Reasons for no communication*	
For delivery mother went to parents’ house	100
TBA was at delivery	25
Rich mothers go straight to facilities	7.4
No complication	7.4
Who communicated**	
Mothers attended orientation session	85.2
Only family members attended orientation session	25.9
Come at father’s house for delivery	44.4
Heard from relative/neighbor	40.7
Motivated mothers by CSBAs	59.3
Motivated mothers by CSG	40.7
Heard from the oriented participants	37.0

* Multiple response and proportion >5% have been shown in table; reasons included only for delivery period or both antenatal and delivery period.

** Multiple response and proportion >25% have been shown in table.

In formative research, most mothers had less accessibility to mobile phone due to husbands’ subscription to mobile phone. Mothers of low income group (<$100) mostly had poor accessibility to the mobile services. At the same time the CSBAs included in the formative research also mentioned about husband’s subscription as a major obstacle for accessibility to mobile services by mothers. Some CSBAs indicated that some husbands do not allow their wives to use their mobile phone. However, irrespective to accessibility, all types of respondents included in formative research perceived that mobile communication would reduce the response time to obstetric emergencies and maternal issues.

### Time of communication during pregnancy

All CSBAs had mobile communication with mothers more than once. Mothers communicated with the CSBAs through mobile phone during delivery and antenatal period, no CSBAs reported about such communication during the postpartum period (Table 2). CSBAs described that due to the intervention mothers became concerned about their illness during pregnancy. This made them responsive to health care over mobile phone during antenatal period. Mothers’ mobile calls to CSBAs were for less movement of fetus, headache, high blood pressure, vomiting, edema, and hemorrhage during pregnancy, abdominal pain etc. Fever was also a cause for mobile consultation for many instances,

“*Nowadays mothers*’ *perception over fever during pregnancy is clear and they worry about the dangerous effect of fever*, *so they do not delay to call us for this*.”

While in formative research the CSBAs mentioned severe maternal and neonatal complications as common reasons for communication by mothers,

“*If there is any problem*, *then they ran to me as a crazy person*”.

Prior to the intervention the mode of communication was mostly in person. However, after repeated probing the CSBAs mentioned about occasional mobile communication by mothers.

**Table 2:** Time of communication through toll free mobile phone with the CSBAs during pregnancy period

Communication*	**Percentage (n = 27)**
**When did mothers communicate**	
During pregnancy	48.1
During delivery	51.9
During postpartum period	0.0

*Single response elucidated. The question asked on what time during the pregnancy mother’s mostly communicated.

### Response and attitude of CSBAs

While managing maternal health problems over mobile phone, CSBAs highlighted their flexibility in management as per case. Three types of management actions were reported. Consultation over phone seemed adequate for some mothers, personal visit and higher level consultation were necessary in some cases. CSBAs also linked their morality in mobile phone communication for maternal health management:

“*When a mother called at late night it disturbed me. But if I switched off the mobile at night*, *I feel uncomfortable. A mother would fail to inform me if she was in a state of complication at night and she would suffer. So I always put my mobile in a switched on mode*”.

Prior to the intervention the mothers and their husbands who communicated with providers for health issues using mobile phone, experienced some barriers. These included irritability from provider’s side, sometimes the mobile phone found switched off and the network problem especially in case of an emergency. One mother stated:

“*Her mobile was switched off or there was no charge in her mobile*, *in this case the mobile communication would be dangerous for me at the state of emergency*.”

In formative research, most of the mothers and their husbands were concerned about the risk of predicted indirect interaction with providers during health care by using phone. They sensed a major disadvantage of mobile communication: without physical examination, determining the management might affect the appropriateness in curative treatment. One of the CSBAs at that time anticipated that a well established mobile communication pathway might prevent the CSBAs in visiting the mothers. However, at the endline CSBAs were found active in responding mother’s call. CSBAs followed up the mothers to whom she provided ANC by regular phone call or called her few days before the date of delivery. The CSBAs described the ways of their responsibility to follow up,

“*I called back following my advice over phone after passing some times*, *in case of deterioration or no improvement I went to mothers*’ *houses*”.

### Delivery complication and referral

Commonly the TBAs were called first to conduct the delivery and later on CSBAs were called either at the time of complication or for any other problems relating to delivery. This was one of the most important findings in this study. In such a situation, a phone call was made either by the TBA or mother/family member to CSBA. The following case described a situation of mobile communication for a mother, who was in labour.

“*During delivery the TBA pushed several injections but failed to expel the placenta. The family called me over phone. I was attending another delivery*; *at a time it is not possible to attend two deliveries. The family became impatience and was repeatedly calling me. I went there and then succeed to expel the placenta*.”

In maternal complications CSBAs made prompt referral where they were unable to manage. This was reflected when they mentioned the referral process for eclampsia, post partum hemorrhage (PPH), hand prolapsed, first delivery history of caesarian section, short stature height etc. There was either a direct referral following the phone call from mothers or after examination of mothers.

“*The family told me to handle this PPH. I said please forgive me*, *it is out of my way. Please take her to the sub district health facility. But that facility is far away from mother*’*s house and family decided to go to the nearby clinic. I called the doctor of that clinic*, *she prepared the clinic before our arrival. I accompanied the mother and after arrival doctor managed the PPH*”.

“*It was a hand prolapsed*, *when I was informed over mobile phone*, *I told them to go immediately to the sub district hospital. I did not feel confident to deal with this*”.

Mobile communication also helped in seeking care from an appropriate facility without delay. In several instances mothers with complications were referred directly to the district hospital rather than the nearby sub district health complex. These reduced the delay in management by supporting the mothers in receiving the proper treatment. This matched with CSBAs’ and mothers’ perception during formative research on reducing the response time to obstetric emergencies through mobile communication. At the endline one CSBA noted.

“*It was PPH*, *after being informed over phone*, *I visited the mother immediately and referred her to district hospital as because the sub district health facility does not have any blood bank*”.

### Communication between CSBAs and SLG

Majority of the CSBAs (81.5%) communicated with the SLG for expert consultation. The other reasons for communications as reported were maternal complication management and to gain confidence in handling the cases. The principal emergent reason was actually ‘expert consultation’ which seemed to be expressed in various ways. This contact helped in taking decision for referral (59%) or guidance on complication management (55.6%). Although 55.6% mentioned “increase their confidence” again it was a matter of expression which might be related in guiding to referral or managing the complication by CSBAs. Conversely, 20% CSBAs considered such contact non beneficial as because SLG did not respond to their call. However, few CSBAs reported that SLG called back to them. They explained that SLG might not respond instantly to their call due to their engagement in other important tasks (Table 3).

In formative research, consultation between SLG and CSBAs was perceived as a direct benefit by the CSBAs. They expressed that this consultation would be important to increase their confidence in handling the emergencies and make proper decision. The qualitative research at the endline showed mobile communication between CSBAs and SLG members on several events. Communications with the SLG mainly happened at the time of maternal complications. The CSBAs said that such consultation were essential where they have less control over the management. The following quotes revealed that advices from SLG over phone at a critical moment guided CSBAs in managing maternal complication,

“*Mobile phone is like a training provision for me. It seems that a doctor is supervising my work while consulting with the SLG over phone*.” “*Few days before* (*in a case*) *I tried a lot but the placenta was not coming out. The night was progressing*, *at 01.00 am I called one of the members of SLG. I then tried as per her advice and at last the placenta expelled and the mother survived. The family became very happy to me*.”

The following quote is evident about the way of support received from the SLG.

“*Amina apa*, *senior staff nurse*, *commonly wants to know the detail of patient*’*s condition and then advises over phone. She calls back to know the status of the condition of the mothers and after her assessment she gives advice on further action in that regard*, *like referral to the appropriate facility*, *either to the sub district health facility or district health facility*.”.

While in past the SLG was not familiar to receive phone call from a CSBA.

“*In past the situation was not like this. We were afraid to call a doctor by phone. Nowadays*, *it is so easy*, *we can call SLG at midnight and seek for their advice. It is great*.”

Even whenever the SLGs were unable to receive a phone call from the CSBA instantly as for a busy moment, soon they called back to CSBA.

“*For example a member of SLG is conducting a surgery*, *at that moment she is unable to receive my call but they would call me back later. Many times we ring her again. We have eight SLGs*; *we will receive support from at least one of them at anytime*.”

CSBAs reported that at the beginning of this project, mother’s preference was the TBA but during the project implementation period mothers were calling CSBA first. Mother’s differentiation between TBA and CSBA took place because of CSBA’s linkage to SLG. CSBAs described about mother’s thought as following.

“*CSBA has contact with eight doctors. If she cannot contact one*, *there are other doctors. If a doctor is at his*/*her busy moment and cannot answer the phone*, *later on the doctor will call back the CSBA. This phone communication is highly valued*.”

However, CSBAs suggested for more involvement of SLG in this mobile communication. CSBAs also stated the non-cooperative attitudes of some of the SLG as a challenge to their work. They cited instances when the SLG’s phone was switched off, the switched off mainly happened on weekend. In one CSBA’s word.

“*Mother*’*s complication is not based on weekends. The mobile should remain open 24 hours*”.

CSBAs said that they would not disturb SLGs unnecessarily unless they are in need. They suggested for incentive provision for the SLG as a mean of inspiration. They also suggested for monthly meeting between SLG and CSBAs for discussing the success stories and other experiences. Considering the great benefit of this toll free mobile communication in maternal health the CSBAs repeatedly urged for the continuation of this system (Table 3).

**Table 3:** Reasons for communication with SLG by CSBAs through toll free mobile phone and benefit of this communications

**Communication between CSBAs and SLG** *	**Percentage (n = 27)**
**Reasons for communication**	
For expert consultation	81.5
Did not know what to do	29.6
To increase confident	40.7
For complication	88.9
**Benefitted by communication**	
Help to take decision for referral	59.3
Guide to manage the complication	55.6
Increase confidence	55.6
**Reasons for not benefitted by communication**	
Switch off phone	11.1
Did not receive the call	22.2
SLG called back us in response to our call	7.4

*Multiple response.

### Mothers’ view on communication with SLG

The FGD showed that mothers retained basic information about danger signs and obstetric complications that they learnt from the community orientation sessions of this project. Mothers discussed the aspects of the intervention that benefitted them in managing their obstetric and neonatal complications. Mother’s view about communication between SLG and CSBAs was expressed as,

“*Sometimes my mother thought why CSBA is continuously relaying the details of her daughter*’*s condition to the SLG. She should take rapid action and cure me rapidly. She knows that she is talking like an unconsidered mother*, *we are illiterate and she* (*CSBA*) *had the training*, *so she knows how to deal with complication. Actually her consultation with the SLG means she is dealing it carefully. The mobile is advantageous to us at every step*”.

All the mothers of the FGD were adjacent to their respective CSBA residence and they were more comfortable in calling her in person. Therefore, the first loop of mobile communication which was between mother and the CSBAs was not common in this locality. Mothers mentioned that mobile communication occur between mothers and CSBAs who are largely apart by their residence.

For some conditions, such as severe abdominal pain, problem in neonate’s defecation, high blood pressure etc. basic care by CSBA were found sufficient. However, at some point these conditions also needed consultation with SLG.

“*My blood pressure increased just after delivery. The CSBA put running water on my head and in between she called the Big Sir* (*SLG*). *He told her to push an injection and after that injection I recovered*”.

Implicit advantage of a toll free mobile communication was availability of important services to mothers every day at any hour. One mother needed emergency health care at midnight; her husband had a long consultation with the CSBA over phone and CSBA’s advice supported him to manage his wife for the night. In another case mother could avail the CSBA’s service at her home through a midnight phone call,

“*Complication doesn*’*t raise as per time. It appeared at midnight*, *I called the CSBA through mobile and she came immediately*.”

In the union where the FGD was conducted, traveling to the referral health facility is difficult. The distance and transport were substantial for the urgent access to the emergency obstetric care (EmOC). Due to lack of transport, the community managed with the existing vehicles for traveling to EmOC,

“*We prepared a* ‘*macha*’ (*kind of stretchers*); *four people need to carry this with the mother. It is made by bamboo or a flat platform of wood. At the time of danger there is no lack of people to carry this*”.

In some instances mothers felt that the toll free mobile communication between the CSBAs and SLG is bridging the gaps of the long distance in maternal health management.

“*I am here and the big sir* (*SLG*) *is in another place. I didn*’*t need to cross the river. Sitting in a long distance I am receiving treatment through mobile*”.

Among the eight participatory mothers in this FGD, five mothers experienced complications either maternal or neonatal. A question asked on referral for the complication and compliance to referral. The mothers perceived that mobile communication between the CSBA and SLG helped their referral compliance and thus to receive the proper treatment. The mothers were highly confident about the referral center just for the communication with SLG beforehand by the CSBAs.

“*We may go to an unknown doctor*, *who doesn*’*t know anything about me. But through the mobile communication*, *the doctor became aware about my condition beforehand. After reaching the facility we are receiving rapid management*.”

## Discussion

This paper had taken the opportunity of the availability of formative research results to generate a qualitative comparison; while the effect of this toll free mobile communication will be shown through comparing the results between the baseline and endline surveys. This comparison was a mapping review of information in two different time periods rather than a true relationship. This comparative review was suggestive about the attitude and behavioral factors that influenced the mobile phone communications for maternal health problems after the intervention.

The findings from the endline qualitative results showed that 90% CSBAs reported about mother’s phone call to them for maternal health issues whereas in formative research, occasional phone call by mothers was stated. A gender differential for mobile communication among the low income group was revealed in the formative research. Globally, a woman is 21% less likely to own a mobile phone than a man and this is highest in South Asia, followed by Sub- Saharan Africa. Cost was identified as one of the main reasons for not owing a mobile phone [16].The formative research found that lack of access to a phone by mothers of low income group was an effect of husbands’ subscription and also non sharing attitude with their wives. A case study in Uganda showed that the traditional gender roles limit mother’s access to mobile phones and thus affect their quality of life [23]. The introduction of the toll free mobile phone communication in this study could be viewed as a support to reduce this gender disparity. Furthermore, highest proportion of mothers who attended the orientation sessions with their families utilized this communication network. The orientation sessions also reiterated that necessary information promoted mothers to be overcautious during their antenatal period and delivery. This was reflected in the frequency of mobile contact with the CSBAs for the reported complications during pregnancy and delivery.

The formative research results suggested that irritability from the provider’s side, switched off and inadequate network affected the response at maternal emergencies who tried to communicate with providers using phone. The inadequate response from provider’s side might lead to be skeptical or feeling uncomfortable in mobile communication by mothers. The endline qualitative results put forward a change in this attitude. After the intervention the CSBAs used switched on mode, advised the mothers over phone and visited mothers at household level following the mobile communication. Many mHealth studies have documented the effort of text messaging to improve the implementation of health promotion initiatives [25] that might be overwhelming in this direct mobile communication network. In addition, the household visits by CSBAs or immediate referral following a mobile communication could remove fears and concerns of misappropriation in maternal health complication management while health care is based only on mobile communication. Therefore, the uniqueness of this intervention was direct contact between mothers and providers through toll free mobile communication; whereas projects on mhealth have a common limitation for such interaction [16]. Face to face interaction in healthcare was preferred over SMS messages as because SMS messaging often lack of individual “tailoring” [17]. The individual tailoring was achieved in this communication network.

The perceived advantage in reducing response time to obstetric emergencies and maternal issues that emerged in formative research was a fact after the intervention. The study results revealed that CSBAs referred the maternal complication without delay to an appropriate referral health facility. Significant impacts had also shown on remote medical monitoring through mobile medical information system in other studies [24]. Moving from one referral center to another until reaching the proper health facility might incur the cost for travel as well as delay in management; therefore timely referral reduced the response time in the maternal complication management. On the other pathway that is mobile communication with the referral centre by the CSBAs supported the referral compliance. These communications were seen as clinical importance by mothers, initial case information from a respected community level provider would be more acceptable to clinicians of the referral centre and thereafter would influence the rapid response to case management.

The other important perceived advantages of mobile communication were higher level consultation and guidance to the community level skilled providers. In Indonesia, a similar study revealed some specific benefits [26], which are congruent with this perceived advantages. For example mobile phone caused easy communication with supervisors, assistance over phone during complication and coordinated visits thus increased time efficiency [26]. Consultation with specialized health professionals by community based providers and mothers usually do not happen in Bangladesh. At the end of the intervention the results showed that the toll free mobile communication facilitated community level provider’s access to specialized health management when higher level consultation was deemed necessary. On the other hand the consultation with the expert group was seen as a form of proper management from a trusted source. Maternal complications management by a community based skilled provider found to be more acceptable and valued to mothers and their families if they are guided by a senior and expert management and this guidance took place commonly in this intervention. Facilitating the consultation between nurse mid-wives and physicians found important by some other studies; the strategic importance of information and communication technology are mobilizing assistance in extreme cases during home deliveries [27]. Nonetheless, CSBAs themselves were also benefited from expert provider’s guidance through mobile communications in difficult deliveries. This benefit might also change community level provider’s behavior towards the adherence to standard treatment guidelines and hence to provide quality of care.

The study results showed rapid access to expert group management through this mobile communication network for the mothers of the FGD whilst residing in distant villages, having limited access to transport and other skilled provider’s services. Distance and time taken to travel to health facilities prevents many people, especially the poor rural mothers from accessing maternal health services; because the direct cost of transport contributes a substantial proportion of expenditure on health care [7]. Many mothers in this sub district are living in poverty, with poor road infrastructure and transport options and these poor pregnant mothers might miss or delay the opportunity to seek care from a skilled provider. Nevertheless toll free mobile communication encouraged community health providers in remote areas to initiate and provide health services to vulnerable mothers in need with the support of an expert group. This supported a poor family in saving their medical care expenditure for visiting a health facility that includes transport cost, provider’s fees, medicine etc. in many manageable instances (Table 4).

**Table 4:** **Changes from formative research and endline qualitative research results** (**based on Roger**’**s theory**)

**Themes of changes**	**Formative research**	**Endline qualitative research**
Concern (predisposition)	Mothers and their husbands were concerned about the risk of not interacting directly with the providers when using mobile communication	Increased interaction with the providers through mobile eliminated the concern of not interacting directly
Attitude (predisposition)	Irritability from the provider’s side was the most common barrier for accessing health services through mobile phone	CSBAs were more active in responding mothers’ call
Access (infrastructure)	Husband’s ownership over the mobile was the main cause for poor accessibility of mothers to mobile services	Toll free mobile communication made services available at any hour of a day and every day of the week. Mothers didn’t require to wait for their husband anymore
Advantage on mothers’ mobility (Perceived advantages)	Lack of accessibility to maternal services were major problems in hard to reach areas during complications	For first stage complication management; communication between CSBA and SLG accelerated the complication management procedure
Awareness and mode of communication (Adoption)	Occasional mobile communication by mothers but it was not the norm	Increased mobile communication between CSBAs and mothers for maternal health problems

“↓” : Concern, attitude, access and advantage are leading to adoption of mobile phone communication. The results of formative research and endline relating to concern, attitude, access and advantage are causing ‘Occasional mobile communication by mothers but it was not the norm’ and ‘Increased mobile communication between CSBAs and mothers for maternal health problems’ respectively.

## Conclusion

In conclusion, the qualitative results in two different situations facilitated in getting a view of what worked and just as importantly, how it worked. The use of mobile technology in this intervention took a leap from simply rendering information to providing more timely services. The achievement of such initiative was not only dependant on the acceptability and accessibility by the mothers and family but also on the willingness and participation of the service providers at community as well as referral facility level.

The first limitation of this paper was that the results presented here are based on qualitative research. The results of the in-depth interview might be affected from selection bias. Secondly, the study was conducted in one sub district and the mothers of the FGD were from only one remote union, therefore the study was not generalizable.

However, this study has an important implication nationally as because recently the Government of Bangladesh is integrating innovative technology in the health infrastructure for the better response to health needs. This study could recommend that the Government’s innovative technology initiative should take into consideration a provision for immediate and local responses to health problems rather than only information dissemination. This provision should integrate the information dissemination and connecting service according to the need of the people. This study demonstrated that an innovative mobile communication pathway can reduce pregnancy panic in a remote rural area and thereby save money, time and lives of mothers and their neonates. Therefore, further assessment will be needed for introducing toll free mobile communication nationwide.

## Abbreviations

ANC: Ante natal care; CSBAs: Community skilled birth attendants; CSG: Community support group; EmOC: Emergency obstetric care; FGD: Focus group discussion; IRB: Institutional Review Board; icddr, b: International Centre for Diarrheal Disease Research.; MMR: Maternal mortality ratio; MNH: Maternal and neonatal health; PPH: Post partum hemorrhage; SLG: Solution linked group; TBAs: Traditional birth attendants; WHO: World Health Organization.

## Competing interest

The authors declare that they have no competing interests

## Authors’ contributions

Nafisa Lira Huq participated in the proposal development of the study, conducted the qualitative component of the study and drafted the manuscript. Asrafi Jahan Azmi was responsible for implementing the study, data analysis and helped to draft the manuscript. MA Quaiyum participated in the design and implementation of the study and helped to draft the manuscript. Shahed Hossain was the Principal Investigator of the study; he conceptualized the study, coordinated all the activities of the study and guided to finalize the manuscript. All authors read and approved the final manuscript.

## Authors’ information

Nafisa Lira Huq, MSc, BDS Assistant Scientist Centre for Reproductive Health, International Centre for Diarrheal Disease Research, Bangladesh (icddr, b). Asrafi Jahan Azmi, MPH, MBBS Research Investigator. Centre for Reproductive Health, International Centre for Diarrheal Disease Research, Bangladesh (icddr, b). MA Quaiyum, MBBS. Associate Scientist Centre for Reproductive Health, International Centre for Diarrheal Disease Research, Bangladesh (icddr, b). Shahed Hossain, MSc, MBBS, Associate Scientist. Centre for Equity and Health Systems, International Centre for Diarrheal Disease Research, Bangladesh (icddr, b).
